# Empirical evidence of delays in diagnosis and treatment of pulmonary tuberculosis: systematic review and meta-regression analysis

**DOI:** 10.1186/s12889-019-7026-4

**Published:** 2019-06-25

**Authors:** Segun Bello, Rotimi Felix Afolabi, David Taiwo Ajayi, Tarang Sharma, Deborah Olamiposi Owoeye, Omobola Oduyoye, Joseph Jasanya

**Affiliations:** 1 Department of Epidemiology and Medical Statistics, Faculty of Public Health, College of Medicine, University of Ibadan, Ibadan, Nigeria; 2 Cochrane, Editorial and Methods Department, Cochrane Central Executive, Cochrane Informatics and Knowledge Management Department Denmark ApS, c/o Rigshospitalet, afd. 7811, Blegdamsvej 9, 2100 Copenhagen Ø, Denmark

**Keywords:** Pulmonary tuberculosis, Evidence, Meta-regression model, Delay, Patient, Doctor, Treatment, Health system, Total, Disease control

## Abstract

**Background:**

Delays in diagnosis and treatment of pulmonary tuberculosis are a major set-back to global tuberculosis control. There is currently no global evidence on the average delays thus, the most important contributor to total delay is unknown. We aimed to estimate average delay measures and to investigate sources for heterogeneity among studies assessing delay measures.

**Methods:**

Systematic review of studies reporting mean (± standard deviation) or median (interquartile range, IQR) of patient, doctor, diagnostic, treatment, health system and/or total delays in journal articles indexed in PubMed. We pooled mean delays using random-effects inverse variance meta-analysis, investigated for variations in pooled estimates in subgroup analyses and explored for sources of heterogeneity using pre-specified explanatory variables.

**Results:**

The systematic review included 198 studies (831,724 patients) from 78 countries. The median number of patients per study was 243 (IQR; 160–458) patients.

Overall, the pooled mean total delay was 87.6 (95% CI: 81.4–93.9) days. The most important and largest contributor to total delay was patient delay with a pooled mean delay of 81 (95% CI: 70–92) days followed by doctor’s delay and treatment delay with pooled mean delays of 29.5 (95% CI: 25.9–33.0) and 7.9 (95% CI: 6.9–8.9) days respectively. There was considerable heterogeneity in all pooled analyses (I^2^ > 95%). In the meta-regression models of mean delays, studies excluding extra-pulmonary tuberculosis patients reported increased mean doctor’s delay by 45 days on average, non-use of chest x-ray and conducting studies in high income countries decreased mean treatment delay by 20 and 22 days on average, respectively.

**Conclusion:**

Strategies to address patients’ delay could have important implications for the success of the global tuberculosis control programmes.

## Background

Tuberculosis is one of the three major diseases of poverty alongside Malaria and Human Immunodeficiency Virus infection ravaging resource-limited settings [1]. It is the ninth leading cause of death worldwide and the leading cause of death from a single infectious agent, recently overtaking HIV in 2016 [2]. Tuberculosis is of public health significance worldwide and TB-related mortality accounted for US$616bn loss globally between 2000 and 2015; a further projected loss of US$ 984bn has been estimated for the period 2015–2030 if no serious action is taken to reduce disease burden [3]. Also about US$ 7bn is expended annually on care and prevention [2].

Tuberculosis typically presents with non-specific symptoms that may be easily confused with other prevalent febrile illnesses at the early stages of the disease. Thus, disease could progress for weeks or even months before patients contact the healthcare systems. Early phase symptoms are usually non-incapacitating and compatible with day-to-day activities until they are severe enough to warrant concerns by which time several close persons may have been exposed. Health workers’ index of suspicion may also be low especially in settings where the disease is not endemic.

Early diagnosis and prompt treatment are crucial to breaking the chain of transmission of *Tubercle bacilli* in the community. Unfortunately, tuberculosis control programmes largely rely on passive case detection [2] which is heavily dependent on patients’ health seeking behaviour. Delays in the diagnosis of open (infective) cases of pulmonary tuberculosis (PTB) promote continued infection transmission. Every unidentified case, as well as every delayed diagnosis, is a missed opportunity to arrest disease spread. Delays also increase the chances of complications and mortality in the index patient [4, 5]. A major treatment aim involves getting every open case of PTB to attain sputum conversion as soon as possible after diagnosis.

Enormous literature exists on delay measures in diagnosis and treatment of PTB, emphasizing the public health challenges of sub-optimal patient health-seeking behaviour and deficient healthcare systems’ approaches in attaining early diagnosis and prompt treatment of every case of PTB. This is a major set-back in tuberculosis control.

Assessing evidence for delays in the diagnosis and treatment of tuberculosis is of public health significance. Previous systematic reviews have described various delay measures [6], or factors associated with delays, [7–9] or extent of delays [10]. For example, Steeramareddy et al., studied only India literature and reported a descriptive summary of delays but not a quantitative synthesis [6]. Similarly, Storla et al. systematically reviewed and narratively reported studies that assessed patient-level factors influencing delays in diagnosis and treatment of tuberculosis but not study-level factors that could explain variations in delay measures between-study [8]. We are not aware however, of any meta-analysis estimating the average delays in the diagnosis and treatment of PTB at critical points in TB control. This is important to demonstrate the weakest link in the control chain. This study was therefore, conducted to estimate the average delay measures in the diagnosis and treatment of pulmonary tuberculosis. The secondary aim was to investigate for sources of heterogeneity among studies assessing delay measures.

## Methods

This systematic review was registered with PROSPERO number 42016049558.

### Study eligibility criteria

We included studies that measured any delay in the diagnosis or treatment of pulmonary tuberculosis and reported mean (plus standard deviation) or median (plus interquartile range). We were interested in six critical delays which had been described previously [6–8, 10]. *Patient’s delay* was defined as the time lag from first symptom onset to first visit to a qualified doctor or health facility; *doctor’s delay* as the time lag from first visit to a qualified doctor/health facility to diagnosis of pulmonary tuberculosis; *diagnostic delay* as the time lag from first symptom onset to the diagnosis of pulmonary tuberculosis; *treatment delay* as the time lag from diagnosis of pulmonary tuberculosis to the first treatment initiation; *health system* delay as the time lag from first contact with a qualified doctor/health facility to the time of initiation of treatment, while *total delay* was defined as the time lag from awareness of first symptom onset to initiation of treatment [6–10].

### Conceptual framework

**Fig. 1:**
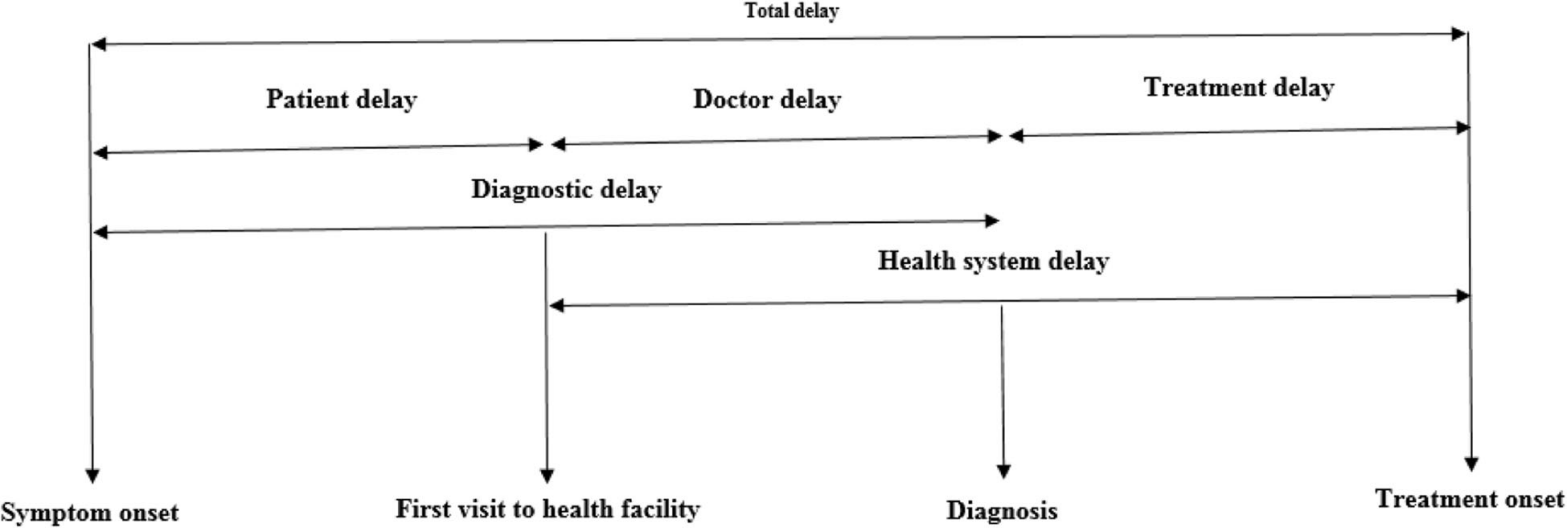
Conceptual framework of delays in diagnosis and treatment of pulmonary tuberculosis

Primary delay measures include patient’s delay, doctor’s delay, and treatment delay. Hybrid delay measures include diagnostic delay which is the sum of patient’s and doctor’s delays; health system’s delay is the sum of doctor’s delay and treatment delay; while total delay is the sum of the three primary delay measures (Fig. 1).

### Search strategy

We developed and implemented a highly specific search strategy in PubMed (Appendix). The core search string included; *“pulmonary tuberculosis” AND delay AND (mean OR median).* The search was conducted on 26 February, 2016.

### Data extraction

Two authors (SB and TS) developed the search strategy and conducted the search. Two authors (SB, DTA) screened titles and abstracts obtained from the database search, and decided on eligibility. We retrieved the full texts of potentially eligible studies. Two authors (SB, DTA, DOO, OO, or JJ) independently confirmed eligibility and extracted data for each record. We extracted data from included studies using a piloted data extraction form. Data extracted included general study background for example study design, study size, country where the study was conducted, year study was conducted, data collection methods, type of health facility where study was conducted, the mean age of study participants, and proportion of study participants that was male. We also extracted data on the clinical description of study participants for example, whether study enrolled only pulmonary tuberculosis patients or whether extra-pulmonary tuberculosis patients were included, whether only smear positive PTB was included and what diagnostic investigation was carried out (sputum microscopy, sputum culture, or chest x-ray).

We also extracted data on the type of delay assessed in the study, author’s definition of the delay, mean (SD) or median (IQR), and range (where applicable).

### Data synthesis

We confirmed author’s definition for each of the delay measures. In some publications, authors used similar descriptive terms for delay measures but gave a definition that differed from the conventional definitions used in this study. For such incompatible definitions, we tried to match them to the appropriate delay that fitted the definition given. For example, some authors used the term diagnostic delay but had given a definition corresponding to doctor’s delay. Here, we recoded this as doctor’s delay. For other definitions that did not match the definition of any of our specified delay measures, we excluded them from the analysis. For studies using the same descriptive terms as ours but without giving any definition, we assumed that their definition was identical to ours. This is because more than 95% percent of studies that used the descriptive terms gave definitions identical to ours. We converted all time measures to days for studies that did not report delay measures in days, by multiplying reported number of weeks by 7 and reported number of months by 30.

We used means and standard errors from studies as primary measure for meta-analysis. We calculated the standard error for study means by dividing the study standard deviation by the square root of the sample size. For studies that reported only median (and interquartile range, IQR), we estimated the mean and SD from the median and IQR [11]. For studies that did not report any measure of variability, we excluded from primary analysis. Time measures in clinical settings are usually skewed but we assumed that the distribution of the study means would approximate the normal distribution, invoking the central limit theorem.

We were aware that some level of clinical as well as methodological heterogeneity would exist between studies such as methods of data collection and clinical characteristics of patients enrolled in studies. Thus, we pooled means for each delay measure from studies, using the random-effects inverse variance meta-analysis. We investigated for variations in pooled estimates by subgroup analysis. We also explored for sources of statistical heterogeneity for delay measures by conducting meta-regression analyses using pre-specified explanatory variables; proportion of study population that is male, mean age of patients, method of data collection (patient record i.e. from routine data, questionnaire survey or both), inclusion of extra-pulmonary tuberculosis (ePTB) in delay measure, PTB type (smear positive or smear negative), use of sputum microscopy, use of sputum culture, use of chest x-ray, whether study was conducted in a tertiary health facility (yes/no), World Bank economic class to which country belongs, World Health Organisation (WHO) region to which country belongs. We also grouped studies according to the 2017 World Bank economic class into high income countries (HIC) and low/middle income countries (LMIC) [12]. Similarly, studies were grouped according to the WHO regions; Africa (AFRO), Americas (AMRO), Eastern Mediterranean (EMRO), Europe (EURO), South East Asia (SEARO) and Western Pacific (WPRO) [13].

If m_*i*_ provides an estimate of the mean for study *i* with standard error for the mean σ_*i*_, the random effects meta-regression allows for residual heterogeneity (between-study variance τ^2^ not explained by the covariates) by assuming that the true means follow a normal distribution around the linear predictor [14]:’
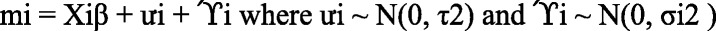


where X_*i*_ is 1 x *k* vector of covariate values in study *i* and *β* is *k* × 1 vector of coefficients.

## Results

### Background characteristics of studies

**Fig. 2:**
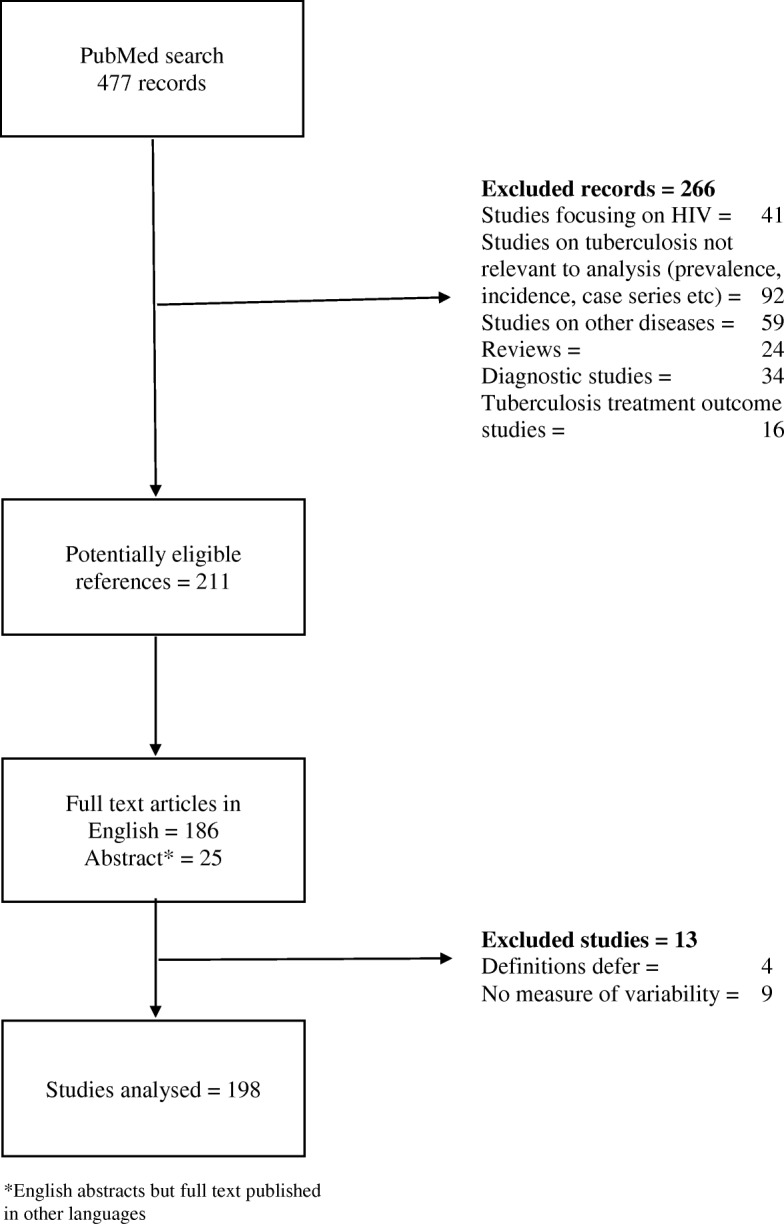
PRISMA flow chart describing reference search and screening

We screened the titles and abstracts of 477 references from database search and identified 211 potentially relevant records consisting of 186 full text study reports published in English and 25 abstracts whose full texts were published in other languages (Fig. 2).

One hundred ninety-eight studies (831,724 patients) from 78 countries were included in this systematic review. Median number of patients per study was 243 (IQR = 160–458) patients. Study size ranged from 13 to 409,152 patients. The year that the studies were conducted ranged from 1983 through 2014; only 43 (21.7%) studies were conducted before 2004, 109 (55.1%) studies were conducted between 2004 and 2014, 46 (23.2%) did not report information on year when data were collected from patients. The reported proportion of males from each study ranged from 35.4 to 100%. More than half (55.0%) of studies reported male proportion of 60% and above. Mean age of participants from studies ranged from 1.75 to 65.2 years. Methods of data collection varied, with 79 (39.9%) collecting data via surveys, 35 (17.7%) collected data from patient records or from a database, 61 (30.8%) used both survey and patient records, no information was provided from 23 (11.6%) studies. Forty studies (20.2%) enrolled ePTB patients in addition to PTB patients. Only smear positive PTB patients were enrolled in 77 (38.9%) studies. Diagnostic investigations used to aid clinical diagnosis included sputum microscopy in 109 (55.1%) studies, chest x-ray in 63 (31.8%) studies and sputum culture in 75 (37.9%) studies.

### Patients delay

Patient delay was analysed in 79 studies (524,462 patients) enrolling a median of 234 patients. Delay ranged from 5 to 1097 days. The pooled mean patient delay was 81 (95% CI: 70–92) days. Two studies from China contributed 92% to the sample size. These were dropped in a sensitivity analysis giving a pooled mean patient delay of 73 (95% CI: 67–79) days.

In subgroup analyses, studies enrolling ePTB with PTB patients reported a pooled mean patient delay (PMPD) of 53 (95% CI 41–64) days compared to a PMPD of 76.0 (63.8–88.2) days for studies not enrolling ePTB patients. Similarly, studies conducted in tertiary centres reported higher PMPD compared to studies conducted in more peripheral centres. Use of only patient records for data collection reported higher PMPD while studies conducted in the two World Bank economic classes reported similar patient delays. Studies from EMRO reported the lowest PMPD compared to AMRO and WPRO which reported the highest PMPD (Table 1). All the differences in subgroup analyses were however, not statistically significant.

**Table 1:** Subgroup analysis of delays in diagnosis and treatment of pulmonary tuberculosis

Study level characteristics	Pooled mean delays in days (95% CI)					
	Patients’	Doctors’	Diagnostic	Treatment	Health System	Total
Plus extra-PTB						
Yes	52.6 (41.0–64.3)	23.6 (13.8–33.3)	58.2 (31.3–85.2)	17.9 (13.6–22.2)	63.5 (37.0–90.1)	80.5 (65.1–96.0)
No	76.0 (63.8–88.2)	24.7 (22.8–26.7)	72.0 (36.2–107.9)	5.7 (4.8–6.5)	37.1 (34.0–40.2)	87.6 (80.3–95.0)
Diagnostic investigation						
sputum mcs	75.0 (67.3–82.6)	26.3 (22.9–33.0)	70.4 (52.8–88.1)	5.2 (3.8–6.5)	37.7 (33.4–42.0)	88.3 (79.1–97.0)
sputum culture	97.0 (71.1–122.3)	27.4 (22.1–32.7)	84.7 (41.4–128.0)	11.6 (10.0–13.3)	43.8 (33.0–54.6)	68.4 (58.8–78.0)
CXR	99.5 (80.3–118.8)	20.4 (15.8–25.0)	45.4 (36.5–54.3)	12.1 (9.8–14.5)	43.5 (32.8–54.2)	97.5 (82.1–112.9)
Sputum smear PTB						
Only smear +ve enrolled	80.2 (62.7–97.6)	46.7 (36.4–56.7)	78.0 (60.8–95.2)	7.8 (6.7–9.0)	36.5 (32.1–40.8)	76.6 (70.8–86.5)
Both +ve/−ve enrolled	93.6 (70.6–116.6)	17.6 (11.9–23.3)	62.0 (2.8–121.0)	8.7 (6.1–11.3)	54.3 (44.9–63.7)	100.7 (87.0–114.4)
Level of healthcare						
Tertiary	87.1 (70.0–104.1)	51.3 (38.6–64.1)	73.0 (36.5–109.4)	30.0 (19.2–40.7)	31.8 (26.7–36.9)	80.0 (70.8–89.5)
Others	75.7 (62.0–89.4)	24.2 (16.3–32.1)	101.3 (75.9–126.7)	7.0 (6.3–9.3)	48.0 (36.3–59.8)	100.9 (87.4–114.3)
Source of data						
Patient record	192.0 (13.0–371.0)	12.0 (11.8–12.2)	38.7 (12.2–65.3)	23.5 (19.9–27.2)	51.9 (29.2–74.6)	65.5 (46.3–84.7)
Survey	58.9 (39.8–78.1)	32.8 (27.9–37.7)	55.0 (9.8–100.2)	3.0 (2.4–3.7)	47.6 (36.9–58.4)	88.8 (79.3–98.4)
Survey + record	82.4 (64.5–100.2)	28.0 (20.6–35.3)	96.8 (70.3–123.3)	4.3 (3.1–5.4)	34.3 (30.4–38.1)	94.9 (81.4–108.4)
World bank class						
HIC	82.2 (62.3–102.1)	17.1 (14.1–20.2)	70.8 (18.4–123.2)	3.5 (2.4–4.7)	63.7 (44.7–82.8)	87.3 (74.1–100.4)
LMIC	83.1 (71.1–95.0)	37.6 (31.4–43.8)	69.2 (59.5–78.9)	9.8 (8.5–11.1)	38.5 (32.5–44.5)	88.4 (80.5–96.3)
WHO regions						
AFRO	68.2 (57.3–79.1)	30.0 (17.3–42.6)	36.0 (22.9–49.1)	7.3 (6.0–8.6)	33.8 (25.9–41.6)	96.0 (79.4–112.6)
AMRO	98.9 (74.9–123.0)	28.5 (22.7–34.3)	132.1 (65.4–198.7)	8.1 (−2.7–18.8)	78.6 (13.3–143.9)	95.7 (78.7–112.6)
EMRO	37.9 (19.7–56.2)	53.3 (37.8–68.7)	70.5 (27.9–113.1)	2.4 (2.1–2.7)	46.6 (29.9–63.3)	85.6 (62.8–108.5)
EURO	71.5 (51.7–91.2)	48.0 (42.1–54.0)	71.9 (19.9–123.9)	17.0 (11.8–22.1)	57.6 (30.6–84.5)	92.7 (61.9–123.5)
SEARO	75.9 (57.1–94.8)	55.3 (30.8–79.9)	53.5 (43.2–63.8)	16.1 (8.8–23.3)	31.7 (13.0–50.4)	82.4 (69.3–95.5)
WPRO	99.8 (81.2–118.3)	15.0 (12.0–17.9)	60.7 (−4–125.7	3.2 (1.6–4.8)	43.2 (22.3–64.1)	71.6 (55.9–87.4)

### Doctors’ delay

Twenty-six studies (499,651 patients) enrolling a median of 247 patients, contributed data to the analysis. The pooled mean doctor’s delay (PMDD) was 29.5 (95% CI: 25.9–33.0) days. Two studies from China and Taiwan accounted for 97.5% of the combined study sizes. In a sensitivity analysis excluding these studies, PMDD was 32.5 (95% CI: 27.8–37.1) days.

In subgroup analyses, studies enrolling ePTB patients reported a PMDD of 23.6 (13.8–33.3) days (Table 1) compared to a similar value of 24.7 (22.8–26.7) days for studies enrolling only PTB patients. Studies using chest x-ray in diagnosis reported lowest PMDD compared to sputum culture and microscopy. Studies enrolling only smear positive PTB reported much higher PMDD compared to studies enrolling both smear positive and smear negative PTB. Also, studies conducted at tertiary centres reported much higher PMDD compared to those conducted at peripheral centres. Studies using only patient record as the only source of data, reported the least PMDD compared to those using survey (Table 1). Studies conducted in World Bank HIC reported lower PMDD compared to LMIC. The least PMDD was reported in studies conducted in the WPRO WHO region followed by AMRO and AFRO.

### Diagnostic delay

Twenty-five studies (416,543 patients) enrolling a median of 253 patients were included in the analysis of diagnostic delay. The pooled mean diagnostic delay (PMDxD) was 69.3 (95% CI 40.1–98.6) days. One study from China constituted 98.2% of the total sample size and was excluded in a sensitivity analysis. The pooled diagnostic delay from the sensitivity analysis was 61.6 (95% CI: 53.4–69.8) days.

In subgroup analyses, studies enrolling ePTB patients in addition reported lower PMDxD. PMDxD was also lowest for studies using chest x-ray compared to studies employing sputum microscopy and culture in diagnosis (Table 1). PMDxD was higher for studies enrolling only smear positive PTB patients, lower for studies conducted in tertiary centres and lowest for studies collecting data from only patients’ records. PMDxD was similar among studies conducted in HIC and LMIC but was lowest for studies conducted in the WHO AFRO, SEARO and WPRO in that order.

### Treatment delay

Thirty-three studies (126,450 patients) enrolling a median of 234 patients were included in the analysis of treatment delay. The pooled mean treatment delay (PMTxD) was 7.9 (6.9–8.9) days. Two studies from Taiwan accounted for 87% of the total study population. Excluding these studies in a sensitivity analysis yielded a PMTxD of 8.4 (7.3–9.6) days.

In subgroup analyses, PMTxD was significantly higher among studies enrolling ePTB patients and also significantly lower among studies using sputum microscopy for diagnosis. However, PMTxD was similar for studies enrolling both smear positive and smear negative PTB patients. PMTxD was also significantly higher among studies conducted in the tertiary compared to the peripheral centres and also significantly higher among LMIC compared to HIC. PMTD was lowest among studies conducted in EMRO and WPRO WHO regions (Table 1).

### Health systems’ delay

A total of 41 studies (28,360 patients) with a median sample size of 253, reported data used for the analysis of health system delay in the diagnosis and treatment of PTB. The pooled mean health system delay (PMHSD) was 39.3 (36.2–42.4) days. One study conducted in China accounted for 36.5% of total patient population. Omitting this study in a sensitivity analysis, gave a pooled mean health system delay of 41.9 (37.3–46.4) days.

In subgroup analysis, PMHSD was significantly higher for studies enrolling ePTB in addition to PTB patients, but only slightly lower for studies employing sputum microscopy compared to sputum culture and chest x-ray. Studies conducted at tertiary centres reported lower PMHSD, while studies that collected data from both survey and patient record reported lowest PMHSD. Studies conducted among LMIC had significantly lower PMHSD. PMHSD was highest for studies conducted in the AMRO region and lowest for studies conducted in the SEARO and AFRO regions (Table 1).

### Total delay

Forty-five studies (32,651 patients) reporting a median sample size of 253, were analysed for total delay in the diagnosis and treatment of PTB. The pooled mean total delay (PMToD) was 87.6 (81.4–93.9) days. One study from China accounted for 31.7% and was omitted in a sensitivity analysis, giving a PMToD of 88.3 (81.6–95.0) days.

The PMToD was similar for studies enrolling only PTB and studies enrolling ePTB patients, but significantly lower for studies employing sputum culture for diagnosis compared to study employing sputum microscopy or chest x-ray (Table 1). PMToD was significantly higher for studies enrolling only smear positive PTB compared to studies enrolling both smear positive and negative PTB patients and also lower for studies conducted in tertiary centres. PMToD was lowest for studies using only patient record as source of data but similar for studies conducted among HIC and LMIC. Studies conducted in the WHO WPRO region reported lowest PMToD (Table 1).

### Meta-regression

More than 90% of the residual variation was due to heterogeneity in all pooled mean delays except for PMDD (78%) (Table 2). The covariate set was able to explain more than 50% of the between-study variance in PMDD, PMDxD, and PMTxD but explained less of the PMHSD than would be expected by chance.

**Table 2:** Meta-regression models of delays in diagnosis and treatment of pulmonary tuberculosis

	Patient	Doctors	Diagnostic	Treatment	HSD	Total
Variables						
Male %	1.4 (−0.8, 3.6)	1.4 (−1.8, 4.6)	5.5 (1.7, 9.3)	−0.09 (− 0.4, 0.3)	−0.3 (− 1.5, 0.9)	−0.8 (−3.2, 1.5)
Mean age	4.1 (−2.7, 10.8)	−4.9 (− 12.4, 2.6)	−6.3 (− 15.6, 3.1)	−0.01 (− 0.3, 0.3)	3.4 (−1.9, 8.8)	7.6 (0.2, 15.0)
MOD	27.5 (− 2.4, 57.4)	−67.6 (− 139.4, 4.3)	117.9 (45.3, 190.6)	− 0.44 (−5.3, 4.4)	0.8 (− 37.5, 39.1)	0.2 (− 56.1, 56.4)
Eptb	55.3 (− 10.5, 121.2)	45.1 (7.9, 82.3)	107.2 (− 15.0, 229.5)	1.44 (−8.3, 11.2)	6.5 (−97.4, 110.5)	35.0 (−77.2, 147.2)
TBtype	− 14.4 (− 42.7, 14.0)	−1.3 (− 22.3, 19.6)	62.9 (− 73.5, 199.4)	0.35 (−3.25, 3.95)	−1.9 (− 28.1, 24.3)	10.9 (− 36.7, 58.5)
Sputum MCS	91.7 (19.8, 163.6)	22.8 (− 13.0, 58.5)	− 4.6 (− 119.1, 109.9)	0.42 (− 7.89, 8.73)	−16.8 (− 78.2, 44.7)	3.1 (− 104.4, 110.5)
Sputum culture	−46.0 (− 116.3, 24.3)	24.6 (− 40.8, 89.9)	23.8 (− 110.0, 157.6)	−0.89 (− 11.8, 10.0)	30.6 (− 34.7, 96.0)	48.9 (− 64.3, 162.2)
Chest X-ray use	−62.9 (− 128.7, 2.8)	179.2 (−9.3, 367.6)	193.7 (− 65.5, 452.9)	−19.72 (− 33.6, − 5.9)	− 6.8 (− 67.2, 53.5)	−31.2 (− 150.1, 87.7)
Tertiary facility	18.6 (−70.3, 107.5)	−19.3 (− 100.6, 62.1)	205.3 (− 35.3, 445.9)	1.74 (− 9.9, 13.4)	−7.0 (− 56.2, 42.1)	−13.1 (− 117.4, 91.1)
World bank class	−118.2 (− 243.2, 6.8)	275 (− 32.4, 582.5)	129.4 (− 134.2, 393.0)	− 22.07 (− 39.5, − 4.6)	29.1 (− 61.9, 120.2)	3.1 (− 150.3, 156.6)
WHO region	−10.7 (− 29.8, 8.3)	9.2 (− 14.6, 33.0)	−33.5 (− 17.1, 4.1)	0.52 (− 2.2, 3.3)	−2.7 (− 23.0, 17.6)	−14.0 (− 44.9, 16.9)
Model parameter						
Tau^2^	4789.0	17.1	2173	5.2	572.7	2426
I^2^ residual	99.4%	71.3%	98.4%	98.79%	93.22%	94.08%
Adjusted R^2^	16.08%	97.7%	62.0%	78.6%	−18.45%	27.19%
Model F	1.52	16.23	3.14	5.60	1.29	1.86
*p*-value	0.19	0.059	0.141	0.055	0.4139	0.1945

Only for PMDD and PMTxD were the remaining between-study variance reduced significantly. Overall test for covariate set was close to statistical significance for PMDD and PMTxD. For PMDD, non-inclusion of ePTB patients in measuring delays increased mean doctor’s delays by 45 days on average (Table 2). For PMTxD, non-use of chest x-ray decreased the delay by about 20 days on average while conducting studies in HIC reduced PMTxD by 22 days on average.

Even though sputum microscopy (in mean patient delay model), proportion of males enrolled and method of data collection (in mean diagnostic delay model), and mean age of patients (in mean total delay model) were statistically significant in the respective models, the overall test for statistical significance of at least one variable in the covariate set was negative (Table 2).

## Discussion

### Main findings

Overall, the pooled mean total delay in the diagnosis and treatment of pulmonary tuberculosis was 88 days. The most important and largest contributor to total delay in the diagnosis and treatment of pulmonary tuberculosis was patient delay with a pooled mean delay of 81 days followed by doctor’s delay and treatment delay with pooled mean delays of 30 and 8 days respectively. The other hybrid delays including diagnostic and health system delays reported pooled mean delays of 70 and 42 days respectively. All pooled mean delays were considerably heterogeneous.

Some study level characteristics may account for some of the extreme heterogeneity found among studies measuring delays. Excluding ePTB patients increased mean doctor’s delay by 45 days on average, non-use of chest x-ray and conducting studies in HIC decreased PMTxD by 20 and 22 days on average, respectively. Other potential factors that may increase delays were: non-use of sputum microscopy in studies assessing patient delay; increased proportion of males enrolled and using survey method of data collection in the assessment of diagnostic delay; and increased mean age of patient enrolled in the assessment of total delay.

### Strength and weaknesses

We searched only PubMed implying a potential for missing studies that had not been indexed in PubMed. We however, believe that the number of studies included in this systematic review more than triple number of studies included in any other systematic review of delays in diagnosis and treatment of PTB and missing studies are likely to have minimal impact on findings from our study.

Our study tested covariates with high variability across studies, making interpretation of the meta-regression easier. However, there is still a potential for ecological bias. For characteristics with both study-level and within-study individual patient-level values, the results from the meta-regression may not translate to patient-level. For example, for every 1 year increase in mean age across studies, we found that there was a corresponding increase of about 8 days in mean total delay. This may not imply that older people were likely to exhibit a greater total delay unless this has been demonstrated within studies. Thus, some caution is warranted in extrapolating our findings in the subgroup and meta-regression analyses to individual patient level.

Furthermore, the lack of statistical significance may not imply a lack of relationship between covariate and means of delays. This may be attributable to study precision even though we have included a fairly large number of studies. Also, the number of covariates that we have included was relatively modest and missing covariate values were infrequent. Covariates were also pre-specified in the protocol thereby, avoiding data dredging.

Our study did not take into consideration co-morbidities for example, HIV infection. Co-morbidities may likely be relevant predictor of delays in the diagnosis and treatment of pulmonary tuberculosis.

### Mechanisms and results in context of other studies

Tuberculosis is a chronic disease that requires a high index of suspicion by both the patient and the healthcare providers to enhance prompt diagnosis. It is conventionally recommended that cough lasting two or 3 weeks should arouse the suspicion of affected person to seek help, [15] and for the healthcare provider to screen by at least conducting a sputum microscopy test. Our study reveals there exist a considerable average delay before patients seek help from the healthcare providers. This may not be unconnected with the insidious-onset nature of the disease. More than 10 weeks on average is wasted before patients consult the appropriate health facilities. In some settings, it could be as a result of self-medication or seeking help from traditional healers or other providers outside the formal health system. Sreeramareddy et al. reported a lower average of 4 weeks for patient delay, [10] which could be accounted for by the differences in method of analysis. The authors presented a descriptive summary which did not weight the studies whereas our meta-analysis provided an average weighted by the inverse variance method. Secondly studies assessing delays that included ePTB was excluded from their primary analysis and thus may partly account for higher PMPD in our study although the influence of ePTB on PMDD remains largely unclear. We had postulated that inclusion of ePTB would likely delay diagnosis but our findings from the meta-regression analysis shows the reverse and supports lower doctors’ delay when other factors were kept constant. However, studies enrolling ePTB were disproportionately (80%) from LMIC which was associated with higher PMDD. Only our study was strict with the definition of delays compared to other systematic review [6–10].

Only for PMToD did mean age of study participants significantly increased the delay. An increase in study participants mean age of 1 year increased the mean total delay by about 8 days. Consistent with our finding were reports of within-study relationship between age and delay measures generally, including total delay. This is possibly a reflection of poorer socio-cultural and economic supports for the older persons in the settings where the studies were conducted. One within-study evidence demonstrated longer health system and total delay for patients older than 60 years [16]; also longer diagnostic and treatment delays for patients older than 65 years was reported in another study [4].

There are however, inconsistencies regarding the influence of gender on delays from patient-level evidence demonstrated in previous studies. Even though an increase of 1 % in the proportion of males was associated with an increase of about 6 days in only mean diagnostic delay, several patient-level studies have demonstrated an association between male sex and longer patient delays, [5, 17, 18] between female sex and longer patient, [19–21] diagnostic, [21, 22] health system, [16, 23] total [20, 21, 24] and treatment delays [21]. Other studies reported no association between sex and patient, [25–29] doctor’s, [21, 26] health system [21, 30] and total delays [26, 31]. These discrepancies may reflect differences in health seeking behaviours and gender roles in different settings.

Inclusion of smear negative PTB patients did not independently account for variations in delay measures. We had postulated that smear negative PTB would likely increase the number of investigations that would be requested before confirming the diagnosis which would increase at least the diagnostic and/or health system delay. This was however, not strongly supported by our findings contrary to findings demonstrated in some patient-level studies. Although, smear negative PTB was associated with an increased pooled mean diagnostic delay of 62 days on average, this was not statistically significant in the model. Patient-level studies demonstrated that smear negative result was associated with diagnostic, [4] and health system delays [30]. However, use of chest x-ray may not have uniform influence on diagnostic delay. It could aid diagnosis and reduce delays in centres with relevant expertise. Only the mean treatment delay was significantly reduced by non-use of chest x-ray in the meta-regression. Some health facilities may not have Chest x-ray. Thus in such centres, diagnosis may be based solely on clinical acumen and sputum microscopy. In contrast to our finding, absence of initial chest x-ray examination was associated with longer health system and total delays in a patient-level study [16].

Depending on the countries, studies conducted in the tertiary facilities may favour longer or shorter delays. For example, being diagnosed in a tertiary health facility may mean that patients had visited lower levels of healthcare delivery previously, implying longer delay. However, it could also mean that the tertiary facilities are more equipped to arrive at the diagnosis and institute treatment within a shorter duration, especially in settings where patients have direct access to the tertiary centres. The level of healthcare delivery did not significantly explain the variations in any of the delay measures but in the subgroup analysis, studies carried out in tertiary centres reported twice the PMDD compared to studies conducted in lower levels of healthcare delivery.

Studies conducted in the HICs were significantly associated with a decreased mean treatment delay. This may be a result of better access to anti-tuberculosis drugs in HICs than in the LMICs. However, Sreeramareddy et al. reported similar patient and health system delays for both high income and low income countries [10]. The WHO regions where studies were conducted did not independently explain variations in the mean delays however there was a tendency for the EURO region to have a higher pooled mean diagnostic, treatment and health system delays than the high burden SEARO and AFRO regions in the subgroup analysis. Clinician’s familiarity with disease presentation and a higher index of suspicion may play an important role in early disease recognition in endemic settings compared to settings where disease prevalence is low.

### Implications

Findings from this study implicates patient health seeking behaviour as the weakest link in attaining early diagnosis of pulmonary tuberculosis. Guidelines recommend that patients with a cough productive of sputum and lasting for two or 3 weeks should arouse a suspicion of pulmonary tuberculosis. The evidence demonstrates a 7 week gap between a target of 3 weeks and the average time it takes patients to turn up at the health facilities for proper assessment. Furthermore, the evidence also implicates the health systems which are expected to arrive at a diagnosis and commence treatment within a week.

The passive case detection strategy may not be optimal for early diagnosis of pulmonary tuberculosis. Considering the mode of disease spread, additional efforts such as high risk screening among high-risk exposed household relatives of index patients may improve early detection rate. Sputum sample assessment of contacts has been shown to detect PTB at an early stage in about 7 % of over a thousand contacts screened in a study [32]. Moreover, the period of infectivity may be much higher than the patient delay because TB prevalence surveys revealed a higher burden of culture-positive or X-ray detected PTB in individuals without symptoms [33]. There is need for further research on cost effective methods to enhance prompt reporting of chronic cough to accessible health facilities. Further operational research for example, on the use of simple and cost-effective algorithms for any cough presenting at health facilities may be warranted.

## Conclusion

Our study provides empirical evidence that demonstrates that the most important primary delay measure in the diagnosis and treatment of pulmonary tuberculosis is patient delay. Even though estimates of delay measures in the diagnosis and treatment of pulmonary tuberculosis vary from one setting to another, some of the variations may be accounted for by differences in variables such as mean age of patient recruited, proportion of study participant composed of a particular sex, use of diagnostic investigations such as chest x-ray and economic status of settings where studies were been conducted. Strategies to improve detection rates and health system efficiency may impact positively on disease control.

## Data Availability

The datasets used and/or analysed during the current study are available from the corresponding author on reasonable request.

## Appendix

### Search Strategy

((pulmonary tuberculosis) OR (koch’s disease)) AND ((total delay) OR (diagnostic delay) OR (doctor’s delay) OR (patient’s delay) OR (treatment delay) OR (health system delay)) AND (mean OR median). Search was conducted on the 23rd of February, 2016.

## Abbreviations

AFRO: WHO Africa Region
AMRO: WHO Americas Region
EMRO: Eastern Mediterranean Region
ePTB: Extra-pulmonary Tuberculosis
EURO: Europe Region
HIC: High Income Countries
IQR: Interquartile Range
LMIC: Low and Middle Income Countries
PMDD: Pooled Mean Doctor’s Delay
PMDiD: Pooled Mean Diagnostic Delay
PMHSD: Pooled Mean Health System Delay
PMPD: Pooled Mean Patient Delay
PMTD: Pooled Mean Treatment Delay
PMToD: Pooled Mean Total Delay
PTB: Pulmonary Tuberculosis
SD: Standard Deviation
SEARO: South East Asia Region
TB: Tuberculosis
WPRO: Western Pacific Region
